# An Unprecedented CeO_2_/C Non-Noble Metal Electrocatalyst for Direct Ascorbic Acid Fuel Cells

**DOI:** 10.3390/nano13192669

**Published:** 2023-09-28

**Authors:** Chenxi Qiu, Qiang Zhou, Rui Gao, Yizheng Guo, Jiaqi Qin, Dongqi Wang, Yujiang Song

**Affiliations:** State Key Laboratory of Fine Chemicals, School of Chemical Engineering, Dalian University of Technology, Dalian 116024, China; chenxi.qiu@xakaili.com (C.Q.); zhouqiang@mail.dlut.edu.cn (Q.Z.); gaorui1994@foxmail.com (R.G.); dagongyizheng@sina.com (Y.G.); qinjiaqi@mail.dlut.edu.cn (J.Q.)

**Keywords:** direct ascorbic acid fuel cells, electrocatalysis, AA oxidation reaction, cerium oxide

## Abstract

Direct ascorbic acid fuel cells (DAAFCs) employ biocompatible ascorbic acid (AA) as fuel, allowing convenient storage, transportation, and fueling as well as avoiding fuel crossover. The AA oxidation reaction (AAOR) largely governs the performance of DAAFCs. However, AAOR electrocatalysts currently have low activity, and state-of-the-art ones are limited to carbon black. Herein, we report the synthesis of an unprecedented AAOR electrocatalyst comprising 3.9 ± 1.1 nm CeO_2_ nanoparticles evenly distributed on carbon black simply by the wet chemical precipitation of Ce(OH)_3_ and a subsequent heat treatment. The resultant CeO_2_/C shows a remarkable AAOR activity with a peak current density of 13.1 mA cm^−2^, which is 1.7 times of that of carbon black (7.67 mA cm^−2^). According to X-ray photoelectron spectroscopy (XPS), the surface Ce^3+^ of CeO_2_ appears to contribute to the AAOR activity. Furthermore, our density functional theory (DFT) calculation reveals that that the proton of the hydroxyl group of AA can easily migrate to the bridging O sites of CeO_2_, resulting in a faster AAOR with respect to the pristine carbon, -COOH, and -C=O sites of carbon. After an i-t test, CeO_2_/C loses 17.8% of its initial current density, which is much superior to that of carbon black. CeO_2_ can capture the electrons generated by the AAOR to protect the -COOH and -C=O sites from being reduced. Finally, DAAFCs fabricated with CeO_2_/C exhibit a remarkable power density of 41.3 mW cm^−2^, which is the highest among proton-exchange-membrane-based DAAFCs in the literature.

## 1. Introduction

Ascorbic acid (AA, vitamin C) can be either directly extracted from green plants or biochemically synthesized [1,2]. Relative to hydrogen and alcohol fuel, AA is much safer for use without fuel crossover and can be easily transported, stored, and refueled [3]. As an advantageous supplementation to hydrogen fuel cells, low-temperature direct AA fuel cells (DAAFCs) have many potential applications in portable chargers, unmanned aerial vehicles, cochlear implants, and pacemakers [4,5,6,7,8]. Since Fujiwara et al. first studied low-temperature DAAFCs [9], many have contributed to this emerging field [10]. However, the development of DAAFCs has been seriously jeopardized by the slow kinetics of the AA oxidation reaction (AAOR) at the anode side. Additionally, proton-exchange-membrane-based DAAFCs only have a record power density of 31 mW cm^−2^ [11]. It is crucial to investigate advanced AAOR electrocatalysts to improve the single’s cell performance.

In recent years, several AAOR electrocatalysts have been investigated, including noble metals [12,13], TiO_2_ [14], polymers [2,15,16], porphyrins [17,18], and high-surface-area carbon black [11,19,20]. Our group previously reported that carbon black (BP2000) is the most effective AAOR electrocatalyst [11]. However, state-of-the-art AAOR electrocatalysts remain as carbon black and deep un-derstanding on the nature of AAOR active sites are still missing. Inspired by the fact that CeO_2_ can electrochemically oxidize methanol [21,22,23], ethanol [24], formic acid [25], and glycerol [26,27], we are curious about whether CeO_2_ can function as a new type of AAOR electrocatalyst after being supported on carbon.

Herein, we report the synthesis of a series of CeO_2_/C electrocatalysts simply by the wet chemical deposition of Ce(OH)_3_ on carbon, followed by a heat treatment to convert Ce(OH)_3_ to CeO_2_ in the air. Typical CeO_2_/C comprises 3.9 ± 1.1 nm CeO_2_ nanoparticles evenly distributed on carbon black. CeO_2_/C exhibits an exceptional activity with an AAOR peak current density of 13.1 mA cm^−2^, which is 1.7 times of that of carbon black (7.67 mA cm^−2^). We found that the CeO_2_, -COOH, and -C=O of carbon and pristine carbon can strongly adsorb AA and are active sites for the AAOR. We investigated the origin of the high level of AAOR activity with X-ray photoelectron spectroscopy (XPS) and a density functional theory (DFT) calculation. Furthermore, after 4 h of an i-t test, CeO_2_/C lost 17.8% of its initial current density, which is much superior to that of carbon black (a loss of 33.1%). We explored the reason why CeO_2_/C has a superior stability to that of carbon black. Eventually, CeO_2_/C was fabricated as the anode of DAAFCs’ single cell, which exhibits the highest peak power density in the literature of 41.3 mW cm^−2^. This study expands our knowledge of the types of advanced AAOR electrocatalysts and presents our understanding of the nature of AAOR active sites for the first time.

## 2. Materials and Methods

### 2.1. Materials

Cerium(III) nitrate hexahydrate (Ce(NO_3_)_3_·6H_2_O, 99%) was ordered from Sigma-Aldrich (St. Louis, MO, USA) and used as received. Potassium hydroxide (KOH, 85%), ethanol (C_2_H_6_O, 99.9%), nitric acid (HNO_3_, 65%), and sulphuric acid (H_2_SO_4_, 98%) were obtained from Sinopharm Chemical Reagent Co., Ltd. (Shanghai, China). Black Pearls 2000 (BP2000) carbon black was purchased from Cabot (Boston, MA, USA). All aqueous solutions were prepared with ultrapure water (18.2 MΩ cm at 25 °C) produced from a Millipore water system (Synergy^®^ UV, Île-de-France, France).

### 2.2. Electrocatalysts Synthesis

Carbon black was treated in 4 M HNO_3_ aq. at 80 °C for 1 h and then washed with deionized water until the pH of filtrate reached 7. The dried carbon black was dispersed in deionized water under ultrasonic agitation, followed by being frozen with liquid nitrogen and lyophilized at −60 °C for 24 h for future use.

In a typical synthesis of 2 wt% CeO_2_/C, 50 mg of treated carbon black was dispersed in 20 mL of deionized water under sonication. Next, 296.5 μL of 20 mM Ce(NO_3_)_3_ aq. was added to the suspension. Subsequently, 3.95 mL of 2 M KOH aq. was added dropwise to the mixture with a pipette under magnetic stirring (300 rpm). After 30 min of reaction, black precipitates were collected by washing them with deionized water until the pH of filtrate reached 7 and dried in a vacuum oven at 65 °C for 1 h. Finally, the sample was heat-treated in a tube furnace at 250 °C for 1 h with a ramping rate of 10 °C min^−1^ from room temperature in air. CeO_2_/C electrocatalysts with different CeO_2_ loadings (1–10 wt%) were also synthesized by simply varying the amount of Ce(NO_3_)_3_ aq. while holding the other reaction parameters constant. The real loadings of CeO_2_ on carbon were determined by thermogravimetric analysis (TGA) and inductive coupled plasma (ICP), which are close to the theoretical values (Figure S1 and Table S1).

### 2.3. Single Cell Tests

An electrocatalyst-coated membrane (CCM) of 4 cm^2^ was fabricated by spraying CeO_2_/C ink with an I/C of 0.1 onto the anode side of a Nafion 211 membrane to reach an anodic electrocatalyst loading of 1 mg cm^−2^. The cathode side was similarly fabricated with commercial Pt/C (JM, 60 wt%) at a platinum loading of 0.6 mg_Pt_ cm^−2^ (I/C = 0.4). Anodic gas diffusion layer (TGP-H-60, Toray, Tokyo, Japan) and cathodic gas diffusion layer (GDL-10d, Sunrisepower Co., Ltd., Dalian, China) were hot-pressed to sandwich the CCM at 130 °C for 2 min to obtain a membrane electrode assembly (MEA). For comparison, MEAs with carbon black as the anodic electrocatalyst were also fabricated under the same conditions. Fuel cell tests were carried out on an in-house fuel cell system. Via a peristaltic pump, 0.5 M AA aq. was continuously delivered to the anode side at a flow rate of 15 mL min^−1^, while humidified oxygen (100% RH) was supplied to the cathode side (400 mL min^−1^). The temperature of liquid fuel, O_2_, and single cell hardware was maintained at 80 °C. Polarization plots were collected using an electronic load (PLZ1004WH, Kikusui, Yokohama, Japan).

### 2.4. DFT Calculation

A pristine 4 × 4 graphene (GRA) layer model was functionalized by hydroxyl, carboxyl, or carbonyl group as well as by the introduction of a Stone–Wales defect. Models of cerium oxide cluster on the GRA were also built.

All models were fully optimized with the B3LYP hybrid functional using Gaussian 09 program [28]. The cerium atoms (Ce) in these models were described by large-core quasi-relativistic pseudopotentials (ECP47MWB for tetravalent Ce and ECP48MWB for trivalent Ce) together with the corresponding basis sets for the valence shells [29,30]. For C, H, and O in the models, the 6–31G(d,p) basis set{#4} was employed [31]. All calculations were conducted in aqueous phase with the solvation effect of water considered through the solvation model based on density (SMD) [32]. The influence of dispersion was considered by Grimme’s DFT-D3 correction in all calculations [33].

Vibrational frequency analysis was carried out to confirm all of the stationary points are minima and to abstract thermal corrections. Gibbs free energies were evaluated at 298.15 K and 1 atm.

The adsorption free energies of AA molecule on GRA ($∆E_{ads}$) were calculated as follows:

ΔG_ads_ = G_GRA+AA_ − (G_GRA_ + G_AA_) (1)

where G_GRA+AA_, G_GRA_, and G_AA_ are the total energies of the GRA with the adsorbate adsorbed, intact GRA, and intact AA molecule, respectively.

The deformation energy of the AA molecule (E_def,AA_) is defined as follows:

E_def,AA_ = E_dis,AA_ − E_eq,AA_ (2)

where E_dis,AA_ and E_eq,AA_ are energies of AA molecule with distorted geometry in adsorbed models and in its intact form, respectively.

## 3. Results and Discussion

This section is divided into subsections. It provides a concise and precise description of the experimental results and their interpretation, as well as the experimental conclusions that can be drawn.

### 3.1. Synthesis and Structural Characterizations of CeO_2_/C

**Figure 1:**
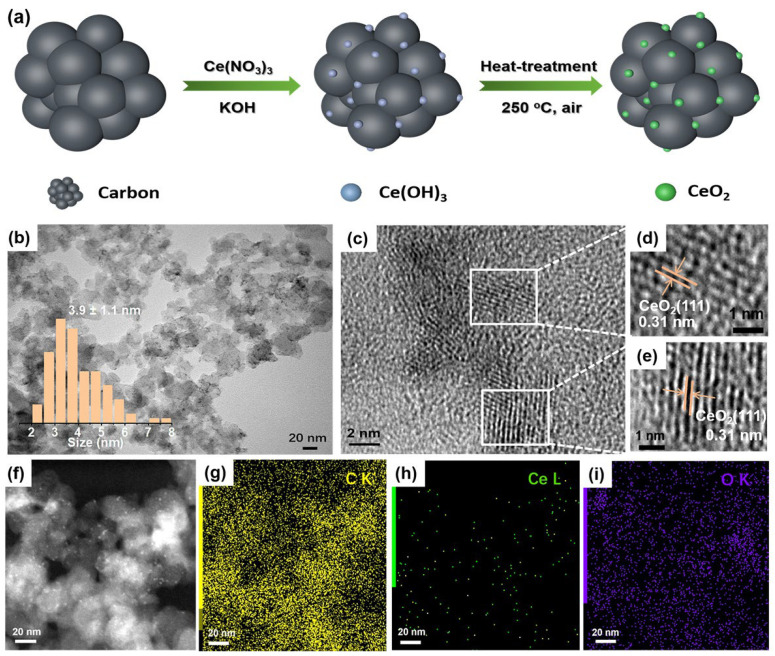
Synthetic scheme and structural characterizations of CeO_2_/C. (**a**) Schematic diagram of synthetic route of CeO_2_/C; (**b**) TEM image of 2 wt% CeO_2_/C; (**c**–**e**) HRTEM images of 2 wt% CeO_2_/C; (**f**) STEM image; and (**g**–**i**) TEM-EDX elemental mapping of 2 wt% CeO_2_/C. Inset: size distribution plot of CeO_2_ nanoparticles created by manually measuring at least 150 individual ones.

In a typical synthesis, carbon black was first mixed with a Ce(NO_3_)_3_ aqueous solution (Figure 1a). Next, KOH aq. was added in to have Ce^3+^ precipitate out on carbon as Ce(OH)_3_. Lastly, the sample was heat-treated at 250 °C in the air for 1 h, during which the Ce(OH)_3_ was oxidized to form CeO_2_ on carbon.

Transmission electron microscope (TEM) images reveal that the CeO_2_ nanoparticles have an average size of 3.9 ± 1.1 nm on carbon without any noticeable agglomerations (Figure 1b). High-resolution TEM (HRTEM) images clearly show that the CeO_2_ nanoparticles are crystalline with a typical lattice spacing of 0.31 nm (Figure 1c–e), which corresponds to the (111) crystal plane of fluorite CeO_2_ [34]. The elemental mapping shows that sparse Ce is uniformly dispersed on carbon in the whole selected area (Figure 1f–i). According to the TGA and ICP results (Figure S1 and Table S1), the loading of CeO_2_ on carbon is 2.15 wt%. The X-ray diffraction pattern (XRD) shows that CeO_2_/C possesses characteristic diffraction peaks of a fluorite structure (Figure S2), consistent with the HRTEM images (Figure 1d,e). According to the Raman spectra, the loading of CeO_2_ did not change the ratio of the D peak to G peak (I_D_/I_G_) of the carbon support (Figure S3), in good agreement with the low-CeO_2_ loading. Similarly, the N_2_ adsorption/desorption measurements in Figure S4 show that the surface area of 2 wt% CeO_2_/C is similar to that of carbon. This implies that the low loading of CeO_2_ barely affects the specific surface area of carbon.

### 3.2. Electrochemical Activity of CeO_2_/C

**Figure 2:**
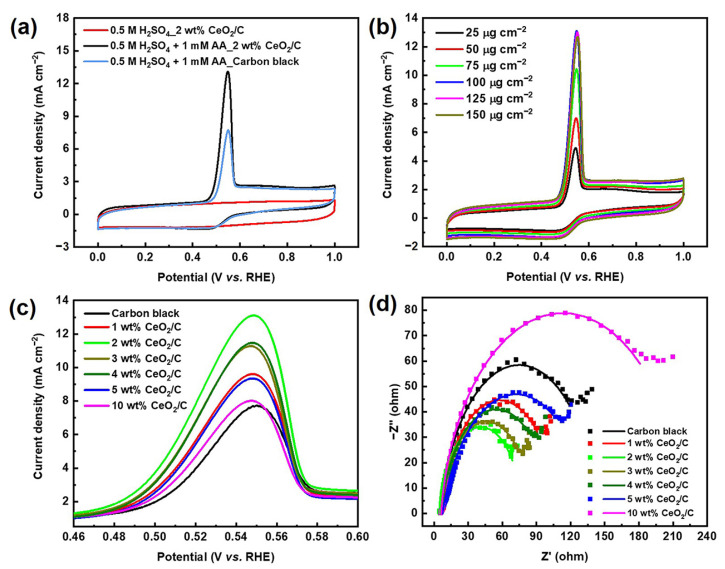
Electrochemical performance of electrocatalysts. (**a**) CV curves of 2 wt% CeO_2_/C and carbon black collected in N_2_-saturated 0.5 M H_2_SO_4_ aq. and 1 mM AA + 0.5 M H_2_SO_4_ aq., respectively, with a sweep rate of 50 mV s^−1^; (**b**) CV curves of 2 wt% CeO_2_/C collected in N_2_-saturated 1 mM AA + 0.5 M H_2_SO_4_ aq. at different loadings of 2 wt% CeO_2_/C on RDE; (**c**) Locally enlarged CV curves of carbon black and CeO_2_/C with different CeO_2_ loadings on carbon collected in N_2_-saturated 1 mM AA + 0.5 M H_2_SO_4_ aq., while keeping the loading of CeO_2_/C on RDE constant at 100 μg cm^−2^; and (**d**) Nyquist plots of carbon black and CeO_2_/C with different CeO_2_ loadings on carbon collected in N_2_-saturated 1 mM AA + 0.5 M H_2_SO_4_ aq. at 0.5 V (vs. RHE).

The cyclic voltammetry (CV) curve of 2 wt% CeO_2_/C exhibits an obvious anodic peak residing at around 0.54 V (vs. RHE) in 0.5 M H_2_SO_4_ + 1 mM AA aq., corresponding to the AAOR (Figure 2a). The AAOR peak current density of 2 wt% CeO_2_/C reaches 13.1 mA cm^−2^, which is about 1.7 times of that of carbon black (7.67 mA cm^−2^, Table S2), which is the previously identified best AAOR electrocatalyst [11,19,20]. Moreover, 2 wt% CeO_2_/C has a lower onset potential of 0.441 V (vs. RHE) compared with that of carbon black. furthermore, we found that the loading of 100 μg cm^−2^ 2 wt% CeO_2_/C on a rotating disk electrode (RDE) resulted in the best AAOR activity (Figure 2b). It is possible that there is a sufficient amount of electrocatalyst and efficient mass transfer at a loading of 100 μg cm^−2^. Higher or lower than this loading, either the amount of electrocatalyst or the mass transfer is not optimal. In addition, the loading of CeO_2_ on carbon apparently influences the peak current density of the AAOR (Figure 2c and Figure S5). It turns out that all of CeO_2_/C electrocatalysts are superior to carbon and the 2 wt% CeO_2_/C has the highest AAOR peak current density. Nyquist plots of the carbon black and CeO_2_/C with different CeO_2_ loadings on carbon show that the 2 wt% CeO_2_/C exhibits the smallest diameter of the half arc, which suggests it has the smallest charge transfer resistance and the fastest reaction kinetics (Figure 2d). This partly explains why the 2 wt% CeO_2_/C has the highest AAOR activity.

### 3.3. Nature of Active Sites and AAOR Mechanism

**Figure 3:**
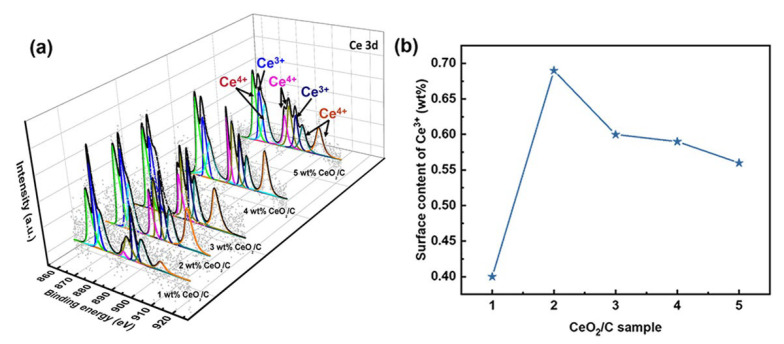
Ce 3D XPS analysis of a series of CeO_2_/C. (**a**) Different CeO_2_ loadings with Ce^3+^ at 886.2 and 904.8 eV and Ce^4+^ at about 883, 888.9, 899.3, 901.9, 908.8, and 917.2 eV; (**b**) surface content of Ce^3+^.

It is necessary to further explore the nature of active sites and the AAOR mechanism. Our X-ray photoelectron spectroscopy (XPS) results show that the percentage of surface Ce^3+^ residing at about 886.2 and 904.8 eV relative to the total amount of Ce species gradually decreases from 34.8% to 20.1% in the series of CeO_2_/C samples (Figure 3 and Table S3) [34,35,36,37,38]. Ce^3+^ and oxygen vacancies co-exist, so Ce^3+^ is usually used to describe oxygen vacancies [39]. As shown in Figure 2c and Figure 3b, it seems that the total Ce^3+^ surface content correlates well to the AAOR activity. Moreover, the 2 wt% CeO_2_/C has both the highest Ce^3+^ surface content of 0.68 wt% and the highest AAOR activity. We propose that the high oxygen vacancy content would be conducive for this improvement in the AAOR activity.

**Figure 4:**
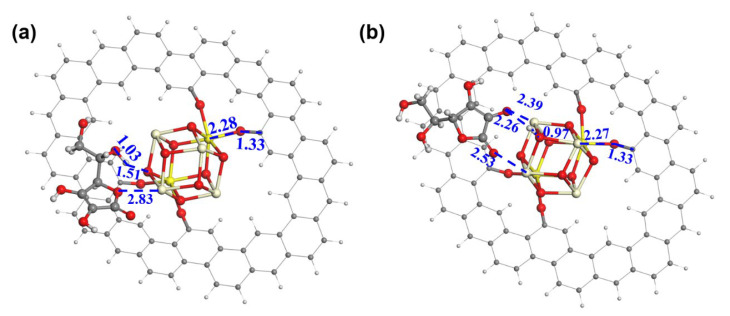
The construction of AA adsorption models on ceria cluster/GRA. (**a**) AA adsorbed on ceria cluster/GRA with the aliphatic hydroxyl and the ether oxygen of the five-membered ring as bridges; (**b**) AA adsorbed on ceria cluster/GRA with two hydroxyl groups of the five-membered ring as bridges. Oxygen, carbon, hydrogen, trivalent cerium, and tetravalent cerium atoms are in red, black, white, light yellow, and bright yellow, respectively. The distances between neighboring atoms are in angstrom.

A DFT calculation was conducted to gain more insight into the role of ceria. In the models (Figure 4a,b), a ceria cluster is anchored on the GRA via four O-bridged bonds. We have also investigated the binding of ceria on GRA via a non-bonded interaction and found it thermodynamically unstable. Hence, the model of the physical adsorption of the ceria on GRA was not considered for the AAOR. The adsorption free energy of AA was calculated to be −40.61 kcal mol^−1^ with the aliphatic hydroxyl group (Figure 4a) and −53.53 kcal mol^−1^ with the hydroxyl group on the five-membered ring connecting to the ceria, respectively (Figure 4b). This strongly suggests that the ceria can provide adsorption sites for AA and thus functions as an efficient AAOR electrocatalyst. Interestingly, when the hydroxyl group of the five-membered ring interacts with the ceria, the proton of the hydroxyl group can easily migrate to the bridging O sites of the ceria. This further implies that the presence of the ceria would accelerate the AAOR process and explains why the 2 wt% CeO_2_/C has an excellent AAOR activity.

### 3.4. Durability of CeO_2_/C and Its Origins

**Figure 5:**
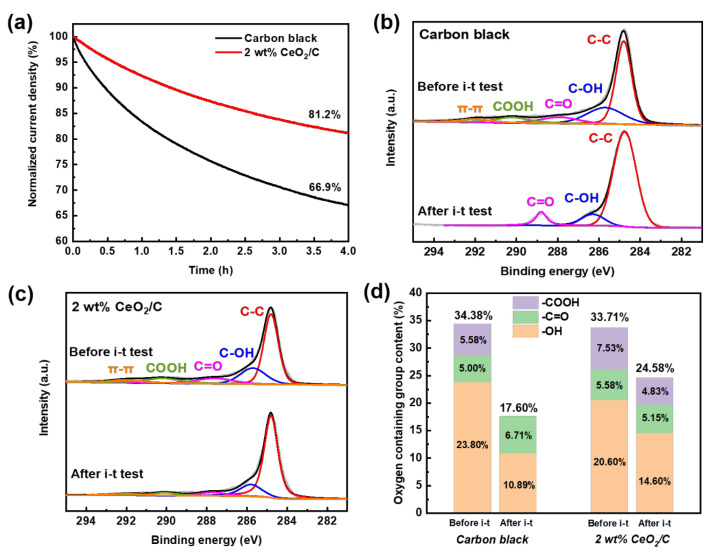
Durability and the origins of CeO_2_/C. (**a**) i-t curves of 2 wt% CeO_2_/C and carbon black collected at 0.5 V (vs. RHE) in 0.5 M H_2_SO_4_ + 1 mM AA aq.; (**b**) C 1s XPS of 2 wt% CeO_2_/C before and after i-t test; (**c**) C 1s XPS of carbon black before and after i-t test; and (**d**) surface oxygen content change of 2 wt% CeO_2_/C and carbon black before and after i-t test based on XPS.

For potential applications, the durability of the 2 wt% CeO_2_/C was also investigated by the chronoamperometric method at 0.5 V (vs. RHE) with carbon black used as a comparison. After 4 h of an i-t test, the current density of the 2 wt% CeO_2_/C degrades by 18.8% (Figure 5a) and is much superior to that of carbon black with a current density degradation of 33.1%. This clearly shows that the 2 wt% CeO_2_/C has an apparently improved durability compared to that of the carbon black. After the i-t test, the surface-oxygen-containing groups of the 2 wt% CeO_2_/C decrease from 33.7% to 24.5%, while those of carbon decrease from 34.4% to 17.6%, as shown in Figure 5b–d and Tables S4 and S5 [11]. It seems that the AAOR activity is closely related to the carbon surface oxygen species. We further employed a DFT to study the nature of the active sites on carbon.

**Figure 6:**
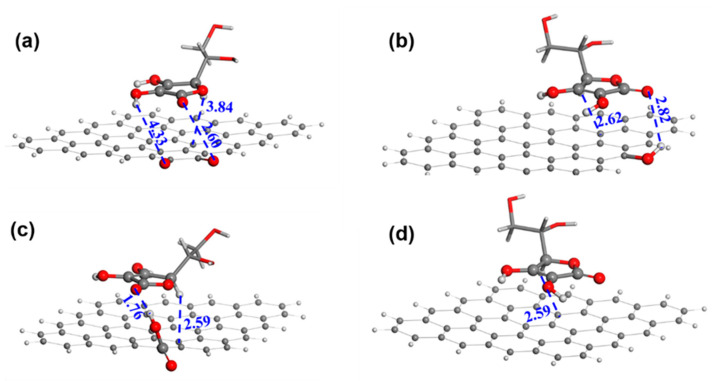
The adsorption geometries of AA on different substrates. (**a**) Carbonyl; (**b**) hydroxyl; (**c**) carboxyl; and (**d**) graphene sheet. Red, black, and white are oxygen, carbon, and hydrogen atoms, respectively. The distances between neighboring atoms are in angstrom.

Figure 6 shows the AA adsorption geometries of GRA with -OH, -C=O, and -COOH. The adsorption of AA at the -OH site is the weakest with an adsorption energy of −1.77 kcal mol^−1^ (Table S6). In contrast, the adsorption at the -C=O and -COOH sites are much stronger with an adsorption energy of −4.48 kcal mol^−1^ and −5.27 kcal mol^−1^, respectively. In addition, the hydrogen bond distance is 1.76 Å between the carboxyl H of GRA and the carbonyl O of the AA’s five-membered ring, while the hydrogen bond distance is 2.82 Å between the hydroxyl H of GRA and the carbonyl O of the AA’s five-membered ring. This is consistent with the corresponding adsorption energy. It is certain that the AA molecule has a higher propensity to be adsorbed and oxidized at the carboxyl site than at the hydroxyl site. Furthermore, the -C=O site and GRA have an AA adsorption energy similar to that of the -COOH site and would also function as AAOR active sites.

During the i-t test, the total content of the -C=O and -COOH of the carbon black decreases from 10.6% to 6.7%. The -COOH has been completely lost with a possible partial conversion to -C=O, which increases from 5.0% to 6.7% after the i-t test. It is more than likely that the -COOH of the carbon black has been gradually reduced by electrons from the AAOR, resulting in the decrease in active sites and AAOR activity. In contrast, the total content of the -C=O and -COOH of the 2 wt% CeO_2_/C slightly decreases from 13.1% to 10.0% (Figure 5d and Table S5), suggesting that the CeO_2_ may have protected the -C=O and -COOH by capturing electrons and thus reducing the loss of active sites. Moreover, our HRTEM images of the 2 wt% CeO_2_/C after the durability test (Figure S6) show that the diameter of the CeO_2_ nanoparticles remains almost unchanged. It is clear that the CeO_2_ nanoparticles are able to survive the i-t test, and the activity loss mainly comes from the degradation of the carbon support.

### 3.5. DAAFC Performance

**Figure 7:**
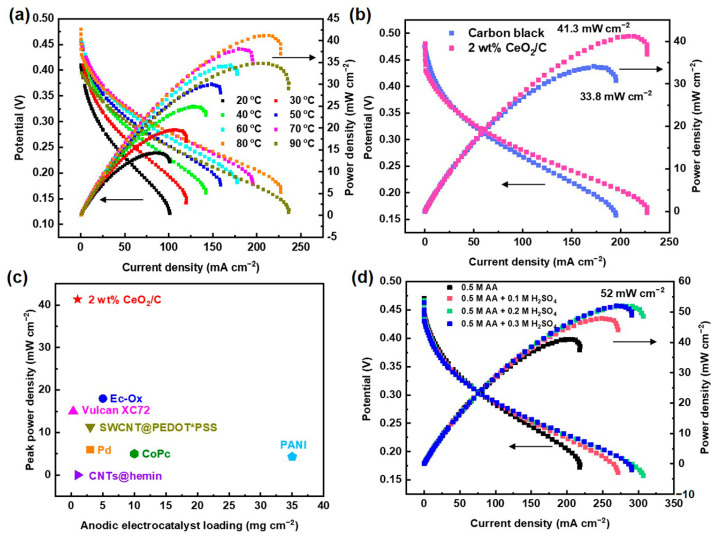
DAAFC performance of 2 wt% CeO_2_/C. (**a**) Polarization curves and power density curves of DAAFCs with 2 wt% CeO_2_/C and carbon black as anodes, respectively, tested at 80 °C; (**b**) polarization curves and power density curves of DAAFCs fabricated with 2 wt% CeO_2_/C as anode tested at 20–90 °C; (**c**) comparison of DAAFCs’ peak power density with different electrocatalysts and loadings; (**d**) polarization curves and power density curves of DAAFCs fabricated with 2 wt% CeO_2_/C as anode, while supplying 0.1–0.3 M H_2_SO_4_ aq. to the anode.

In order to verify the performance of the CeO_2_/C electrocatalyst, 2 wt% CeO_2_/C was fabricated into the anode of a 4 cm^2^ DAAFC single cell with commercial Pt/C (60 wt%) as the cathode. The single cell peak power density reaches 41.3 mW cm^−2^ at 80 °C after temperature optimization (Figure 7a,b). Compared with the single cell fabricated with carbon black (33.8 mW cm^−2^), the power density increases by 22%. This proves that the 2 wt% CeO_2_/C has an excellent activity in single cells. To the best of our knowledge, the peak power density of the 2 wt% CeO_2_/C is the highest among documented DAAFCs using a proton exchange membrane (Figure 7c and Table S7) [2,12,18,19,20,40,41,42]. The peak power density of our DAAFCs with the non-noble metal anodic electrocatalyst is close to that of direct methanol fuel cells typically with PtRu/C as the anodic electrocatalyst [43,44,45]. We suspect that the relatively sluggish AAOR cannot generate enough protons for the cathodic oxygen reduction reaction, thus jeopardizing their single cell performance. Consequently, when an optimal concentration of 0.2 M H_2_SO_4_ and 0.5 M AA aq. was fueled to the anode, the maximum power maximum power density reached 52 mW cm^−2^ (Figure 7d), which is 26% higher than that of the anode fueled without H_2_SO_4_.

## 4. Conclusions

In summary, a series of CeO_2_/C AAOR electrocatalysts were designed and simply synthesized by the wet chemical precipitation of Ce(OH)_3_ and a subsequent heat treatment. Among all documented electrocatalysts, 2 wt% CeO_2_/C exhibits the highest activity with a peak current density of 13.1 mA cm^−2^, which is 1.7 times of that of carbon black (7.67 mA cm^−2^). According to our DFT calculations and XPS experiments, its remarkable activity may stem from its strong AA adsorption on the surface of the ceria and the easy migration of the proton of the hydroxyl group of AA to the bridging O sites of the ceria. After 4 h of an i-t test, 2 wt% CeO_2_/C retains 81.2% of its initial current density, which is much better than carbon black (66.9%). CeO_2_/C merely loses 27.1% of the -COOH and -C=O sites of the carbon support after an i-t test. In contrast, carbon black loses 48.8% of its -COOH and -C=O sites. CeO_2_ may capture electrons to protect the -COOH and -C=O sites from being reduced, thus enhancing durability. Finally, DAAFCs with a 1 mg cm^−2^ 2 wt% CeO_2_/C anode show a maximum power density of 41.3 mW cm^−2^, which is 2.2 times that of the corresponding value in the literature. This study opens up opportunities for the design and synthesis of advanced non-noble metal electrocatalysts for AAORs and illuminates the nature of AAOR active sites for the first time.

## Data Availability

Data can be made available on request.

## Supplementary Materials

The following supporting information can be downloaded at: https://www.mdpi.com/article/10.3390/nano13192669/s1, Experimental: Electrochemical Measurements and Materials Characterizations; Figure S1: TGA curves of carbon black and CeO_2_/C with different CeO_2_ loadings; Figure S2: XRD patterns of carbon black and CeO_2_/C with different CeO_2_ loadings; Figure S3: Raman spectra of carbon black and CeO_2_/C with different CeO_2_ loadings; Figure S4: (a) N_2_ adsorption–desorption isotherms and (b) pore-size distributions of carbon black and 2 wt% CeO_2_/C. Inset: enlarged pore-size distributions; Figure S5: (a) CV curves of carbon black and CeO_2_/C with different CeO_2_ loadings collected in N_2_-saturated 1 mM AA + 0.5 M H_2_SO_4_ aq. Note: the loading of CeO_2_/C on RDE is 0.1 mg cm^−2^; (b) relationship between peak current density and CeO_2_/C with different CeO_2_ loadings on carbon; Figure S6: (a,b) HRTEM images of 2 wt% CeO_2_/C after 4 h i-t test; Table S1: ICP results of CeO_2_/C with different CeO_2_ loadings; Table S2: AAOR performance of carbon black and CeO_2_/C with different CeO_2_ loadings; Table S3: Ce content of CeO_2_/C with different CeO_2_ loadings according to XPS; Table S4: O-containing groups content based on fitted C1s XPS of carbon black before/after i-t test; Table S5: O-containing groups content based on fitted C1s XPS of 2 wt% CeO_2_/C before/after i-t test; Table S6: Calculated adsorption energies of AA (∆G_ads_) on graphene with different oxygen containing groups; Table S7: DAAFCs performance parameters [2,9,18,19,20,40,41,42].
