# Supplementary material for: An Unprecedented CeO2/C Non-Noble Metal Electrocatalyst for Direct Ascorbic Acid Fuel Cells
Source: Nanomaterials (Basel). 2023 Sep 28;13(19):2669. doi: 10.3390/nano13192669 (PMC10574731; doi:10.3390/nano13192669)
Supplement: Supplementary file 1 [file nanomaterials-13-02669-s001.zip › nanomaterials-2607332-supplementary.pdf]

Supplementary Materials

An Unprecedented CeO_2/C Non-Noble Metal Electrocatalyst for Direct Ascorbic Acid Fuel Cells

Chenxi Qiu †, Qiang Zhou †, Rui Gao, Yizheng Guo, Jiaqi Qin, Dongqi Wang * and Yujiang Song *

State Key Laboratory of Fine Chemicals, School of Chemical Engineering, Dalian University of Technology, Dalian 116024, China

Experimental

Electrochemical Measurements

All of the electrochemical measurements were carried out on a CHI 760E electrochemical workstation (CH Instruments, China) in a standard three-electrode electrochemical cell. A graphite rod was used as the counter electrode, and a Hg/Hg_2SO_4 (0.5 M H_2SO_4) electrode acted as the reference electrode. All potentials in this study have been converted to reversible hydrogen electrode (RHE). The conversion parameter of Hg/Hg_2SO_4 to RHE was determined by measuring the voltage difference (ΔE) between the Hg/Hg_2SO_4 and a platinum wire in 0.5 M H_2SO_4 aq. saturated with H_2 at zero current. The ΔE of Hg/Hg_2SO_4 was measured to be -0.7 V in this case. A rotating disk electrode (RDE) with a glassy carbon (GC) disk of 5 mm in diameter was used as the substrate for working electrodes. 2 mg mL^{-1} of ink was prepared by mixing certain amount of an electrocatalyst with water, ethanol and Nafion perfluorinated resin solution (V_{water}:$V_{ethanol}$:V_{Nafion} = 1:9:0.06) under mild sonication for at least 30 min. The ink was pipetted onto the RDE and evaporated in air, resulting in an electrocatalyst loading of 0.025-0.15 mg cm^{-2}. RDE tests were carried out at 25 ^oC in N_2-saturated 0.5 M H_2SO_4 + 1 mM AA aqueous solution. Cyclic voltammetry (CV) curves of an electrocatalyst were recorded from 0 to 1.0 V vs. RHE at a rotation rate of 1600 rpm and a positive scan rate of 50 mV s^{-1}. The durability of electrocatalysts was evaluated for 4h using chronoamperometric measurement at 0.5 V (vs. RHE) in N_2-saturated 0.5 M H_2SO_4 +0.5 M AA aq. at a rotation rate of 1600 rpm.

Materials Characterizations

X-ray photoelectron spectroscopy (XPS) was recorded on an ESCALAB™ 250Xi photoelectron spectrometer (Thermo Fisher, USA) with Al Kα (1486.6 eV) X-ray as the excitation source. Correction of the energy shift was accomplished using the C1s peak at 284.8 eV as the reference. Thermogravimetric analysis (TGA, TA Instruments, Q600, USA) was performed from room temperature to 750 ^oC with a heating rate of 10 ^oC min^{-1} in dried air. High-resolution transmission electron microscope (HRTEM) and energy dispersive X-ray spectroscopy (EDX) were performed on Tecnai G2 Spirit (FEI, USA) operating at 300 kV. Samples for electron microscope analysis were dispersed in ethanol and then dropped on TEM grids. X-ray diffraction (XRD) was carried out on a D/max-2400 (Rigaku, Japan) using Cu Kα radiation source operating at 45 kV and 200 mA. Raman spectra were recorded using a Laser Raman Spectrometer (DXR smart Raman) with a laser wavelength of 532 nm. N_2 adsorption/desorption was measured at 77 K using a Quantacherome Quadrasord-SI Analyzer, where Brunauer−Emmett−Teller (BET) method was used for surface area determination.

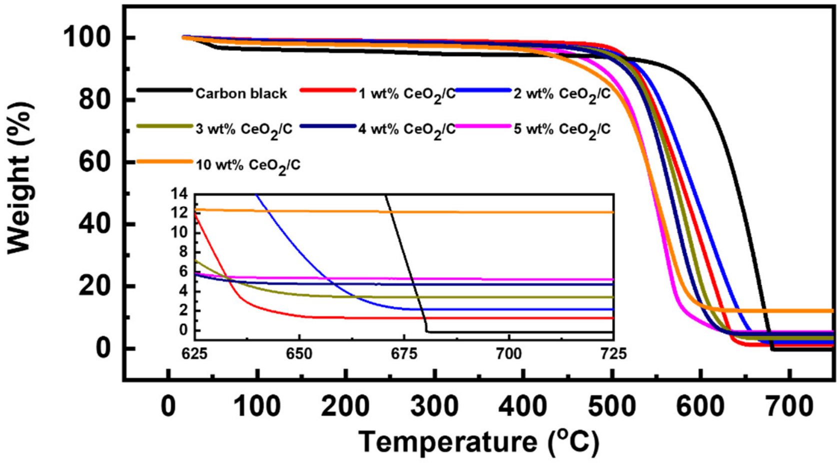

Figure S1. TGA curves of carbon black and CeO_2/C with different CeO_2 loadings.

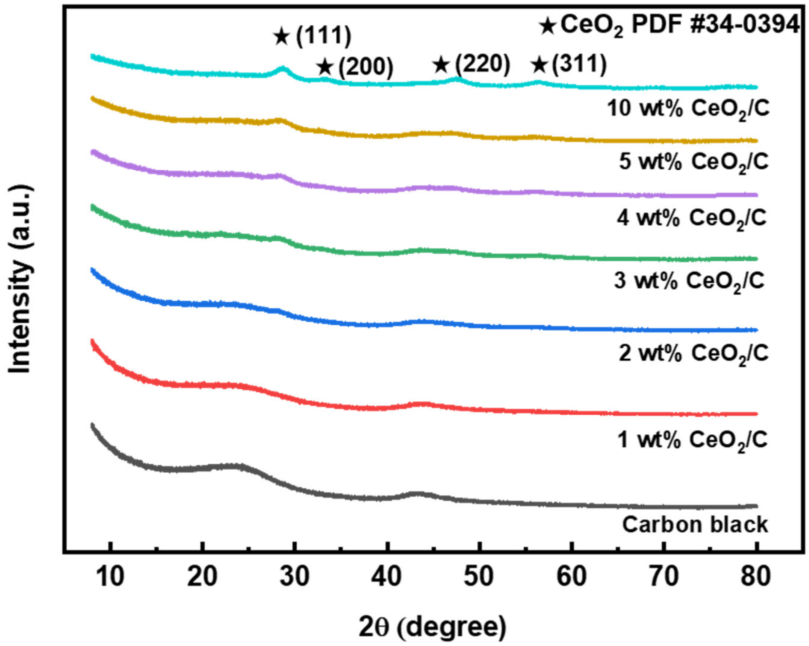

Figure S2. XRD patterns of carbon black and CeO_2/C with different CeO_2 loadings.

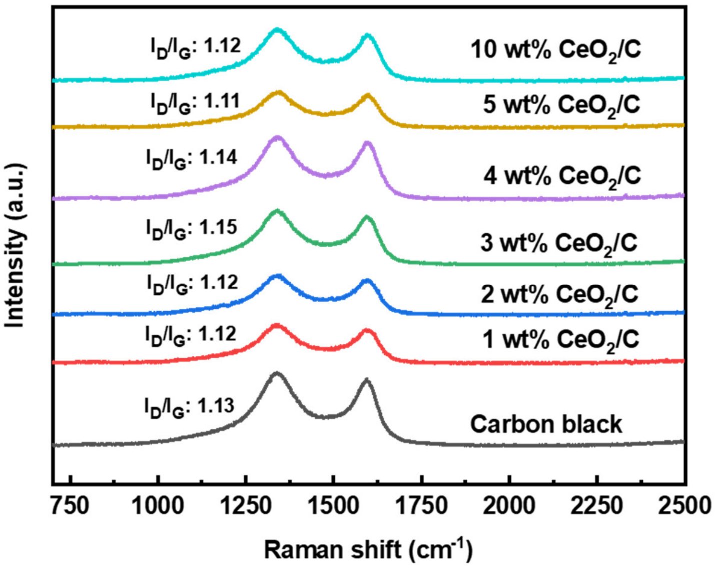

Figure S3. Raman spectra of carbon black and CeO_2/C with different CeO_2 loadings.

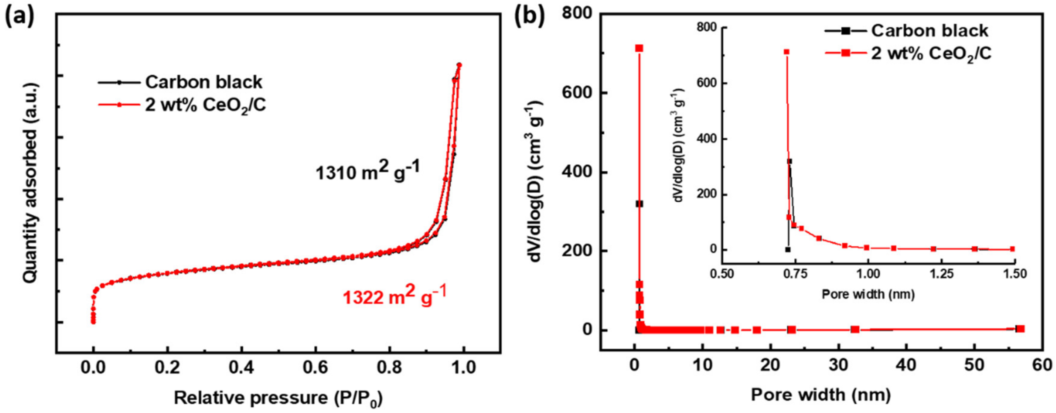

Figure S4. (a) N_2 adsorption–desorption isotherms and (b) pore-size distributions of carbon black and 2 wt% CeO_2/C. Inset: enlarged pore-size distributions.

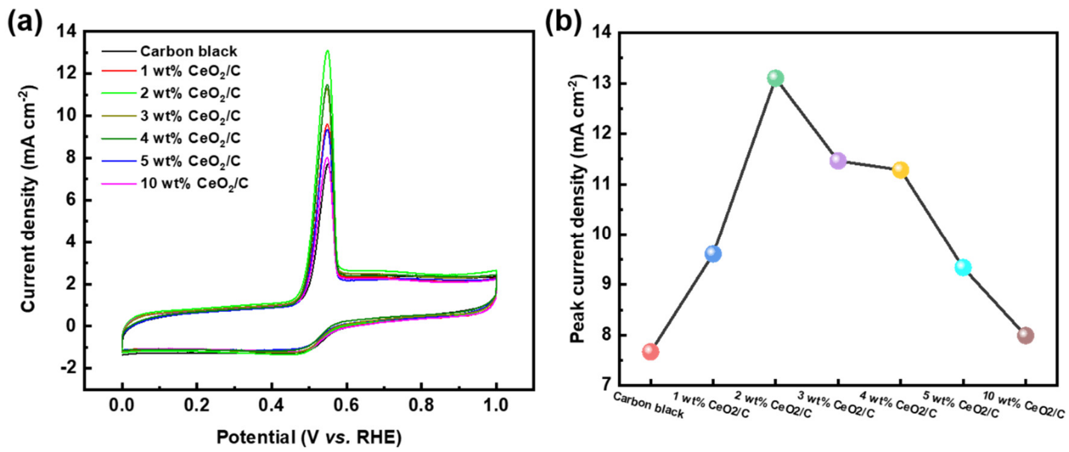

Figure S5. (a) CV curves of carbon black and CeO_2/C with different CeO_2 loadings collected in N_2-saturated 1 mM AA + 0.5 M H_2SO_4 aq. Note: the loading of CeO_2/C on RDE is 0.1 mg cm^{-2}; (b) relationship between peak current density and CeO_2/C with different CeO_2 loadings on carbon.

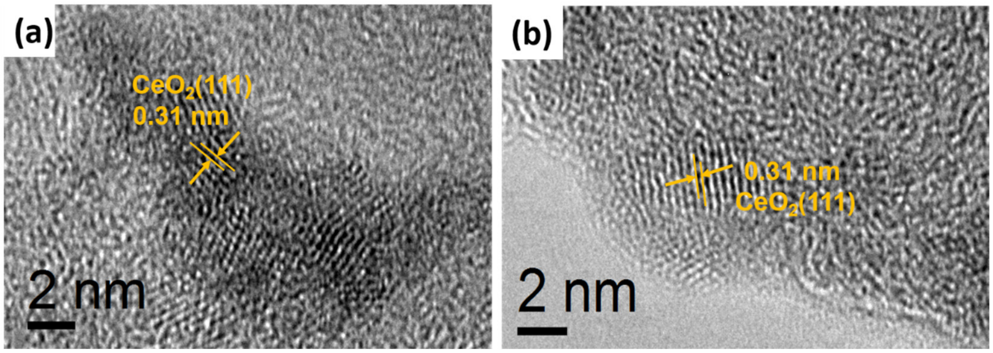

Figure S6. (a-b) HRTEM images of 2 wt% CeO_2/C after 4 h i-t test.

Table S1. ICP results of CeO_2/C with different CeO_2 loadings

Sample	CeO_2 loading (wt%)
1 wt% CeO_2/C	1.28
2 wt% CeO_2/C	2.15
3 wt% CeO_2/C	3.39
4 wt% CeO_2/C	4.71
5 wt% CeO_2/C	5.32
10 wt% CeO_2/C	12.31

Table S2. AAOR performance of carbon black and CeO_2/C with different CeO_2 loadings

Sample	E_{onset} (V vs. RHE)	J_{peak} (mA/cm^2)
Carbon black	0.457	7.67
1 wt% CeO_2/C	0.446	9.61
2 wt% CeO_2/C	0.441	13.10
3 wt% CeO_2/C	0.443	11.46
4 wt% CeO_2/C	0.444	11.28
5 wt% CeO_2/C	0.445	9.34
10 wt% CeO_2/C	0.451	7.99

Table S3. Ce content of CeO_2/C with different CeO_2 loadings according to XPS

Sample	Surface content of Ce	Ce^{3+}	Surface content of Ce^{3+}
1 wt% CeO_2/C	1.15 wt%	34.8%	0.40 wt%
2 wt% CeO_2/C	2.26 wt%	30.8%	0.69 wt%
3 wt% CeO_2/C	2.30 wt%	26.2%	0.60 wt%
4 wt% CeO_2/C	2.40 wt%	24.4%	0.59 wt%
5 wt% CeO_2/C	2.80 wt%	20.1%	0.56 wt%

Table S4. O-containing groups content based on fitted C1s XPS of carbon black before/after i-t test

	-OH	-C=O	-COOH	Total
Before i-t test	23.80%	5.00%	5.58%	34.38%
After i-t test	10.89%	6.71%	0	17.60%

Table S5. O-containing groups content based on fitted C1s XPS of 2 wt% CeO_2/C before/after i-t test

	-OH	-C=O	-COOH	Total
Before i-t test	20.60%	5.58%	7.53%	33.71%
After i-t test	14.60%	5.15%	4.83%	24.58%

Table S6. Calculated adsorption energies of AA (ΔG_{ads}) on graphene with different oxygen containing groups

Name	ΔG_{ads} (kcal mol^{-1})
-C=O	-4.48
-OH	-1.77
-COOH	-5.27
GRA	-4.88

Table S7. DAAFCs performance parameters

Anode eletrocatalyst	eletrocatalyst loading (mg cm^{-2})	Temperature (^{o}C)	OCV (V)	Peak power density (mW cm^{-2})	Reference
SWCNT@PEDOT*PSS	3	80	0.54	11.3	*Chem. Lett.* 2019, 48, 1533-1536[1]
ATC-5	1	35	0.57	0.05	*J. Energy Chem.* 2016, 25, 793-797[2]
CNTs@hemin	1	25	0.65	0.016	*Electrochim. Acta.* 2020, 340, 135946[3]
PANI	35	70	0.50	4.30	*J. Power source.* 2005, 145, 16-20[4]
Ec-Ox	5	60	0.55	18	*Electrochim. Acta.* 2007, 53, 1731-1736[5]
Pd	3	25	0.60	6	*Electrochem. Solid. St.* 2003, 6, 257-259[6]
Vulcan XC72	0.3	25	0.60	15	*Electrochem. Commun.* 2006, 8, 720-724[7]
CoPc	10	25	0.55	5	*Sustain. Energy Fuels.* 2018, 2, 1813[8]
2 wt% CeO_2/C	1	30	0.44	19.4	This work
2 wt% CeO_2/C	1	80	0.48	41.3	This work

References

1. Kaneto, K., Nishikawa, M., Uto, S. Characteristics of Ascorbic Acid Fuel Cells Using SWCNT and PEDOT*PSS Composite Anodes. *Chem. Lett.* **2019**, *48*, 1533-1536.
2. Choun, M., Lee, H.J., Lee, J. Positively charged carbon electrocatalyst for enhanced power performance of L-ascorbic acid fuel cells. *J. Energy Chem.* **2016**, *25*, 793-797.
3. Lin, W.L., Li, N.X., Hu, G.X., Li, H. Hemin-intercalated layer-by-layer electropolymerized co-deposition of bisphenol A on carbon nanotubes for dual electrocatalysis towards ascorbate oxidation and oxygen reduction. *Electrochim. Acta*. **2020**, *340*, 9.
4. Mondal, S.K., Raman, R.K., Shukla, A.K., Munichandraiah, N. Electrooxidation of ascorbic acid on polyaniline and its implications to fuel cells. *J. Power Sources*. **2005**, *145*, 16-20.
5. Uhm, S., Choi, J., Chung, S.T., Lee, J. Electrochemically oxidized carbon anode in direct l-ascorbic acid fuel cells. *Electrochim. Acta*. **2007**, *53*, 1731-1736.
6. Fujiwara, N., Yasuda, K., Ioroi, T., Siroma, Z., Miyazaki, Y., Kobayashi, T. Direct Polymer Electrolyte Fuel Cells Using L-Ascorbic Acid as a Fuel. *Electrochem. Solid-State Lett.* **2003**, *6*, A257.
7. Fujiwara, N., Yamazaki, S.I., Siroma, Z., Ioroi, T., Yasuda, K. Direct oxidation of l-ascorbic acid on a carbon black electrode in acidic media and polymer electrolyte fuel cells. *Electrochem. Commun.* **2006**, *8*, 720-724.
8. Devendrachari, M.C., Thimmappa, R., Bhat, Z.M., Shafi, S.P., Nimbegondi Kotresh, H.M., Kottaichamy, A.R., Venugopala Reddy, K.R., Thotiyl, M.O. A vitamin C fuel cell with a non-bonded cathodic interface. *Sustain. Energy Fuels*. **2018**, *2*, 1813-1819.
